# A chromosome‐level genome assembly of *Solanum brevicaule* (PI 473011) Y1‐5, a wild potato relative with robust resistance to potato cyst nematodes

**DOI:** 10.1002/tpg2.70265

**Published:** 2026-06-12

**Authors:** Senthilkumar Shanmugavel, Shengwei Hu, Huijun Yang, Shiyan Chen, John Bamberg, Dongyan Zhao, Bhoja R. Basnet, Craig T. Beil, Moira Sheehan, Vidyasagar Sathuvalli, Xiaohong Wang

**Affiliations:** ^1^ Hermiston Agricultural Research and Extension Centre Oregon State University Hermiston Oregon USA; ^2^ Plant Pathology and Plant‐Microbe Biology Section, School of Integrative Plant Science Cornell University Ithaca New York USA; ^3^ USDA‐ARS Robert W. Holley Center for Agriculture and Health Ithaca New York USA; ^4^ USDA‐ARS, US Potato Genebank Sturgeon Bay Wisconsin USA; ^5^ Breeding Insight Cornell University Ithaca New York USA

## Abstract

Potato cyst nematodes (PCNs), *Globodera rostochiensis* and *Globodera pallida*, are globally recognized quarantine pests that can cause severe yield losses in potatoes (*Solanum tuberosum* L.) if left uncontrolled. Deploying host resistance remains the most effective, economical, and environmentally sustainable strategy for PCN control. The US Potato Genebank (USPG) maintains a rich collection of wild potato species, providing a valuable resource for discovering novel resistance to PCN. In this study, we identified a *Solanum brevicaule* clone (PI 473011; designated Y1‐5) from the USPG that exhibits robust resistance to both PCN species. We sequenced the whole genome of Y1‐5 using PacBio high‐fidelity sequencing technology and generated a haplotype‐resolved assembly, with genome sizes of 763.49 and 764.93 Mb for haplotypes 1 and 2, respectively. Repeat elements accounted for approximately 65% of the genome, while gene prediction identified 80,021 protein‐coding genes. A comprehensive set of genes encoding nucleotide‐binding and leucine‐rich repeat (NLR) proteins—intracellular receptors that mediate disease resistance—was identified in the annotated genome. *NLR* genes were particularly enriched on chromosomes 4, 6, and 11. Importantly, Y1‐5 was found to encode more coiled‐coil‐type NLRs than several previously sequenced wild *Solanum* clones. These findings suggest that Y1‐5 may serve as a promising new source of resistance to potato diseases. Development of this genomic resource is valuable for understanding the mechanisms of PCN resistance and for supporting potato breeding efforts to develop varieties with durable nematode resistance.

## Core Ideas

- A *Solanum brevicaule* clone Y1‐5 was identified to confer robust resistance to potato cyst nematodes.
- A high‐quality, chromosome‐scale reference genome assembly for Y1‐5 was generated.
- The genome comprises 80,021 predicted protein‐coding genes, including a comprehensive set of genes encoding nucleotide‐binding and leucine‐rich repeat (NLR) proteins.
- Y1‐5 encodes more coiled‐coil‐type NLR (CNL) proteins than several previously sequenced wild *Solanum* clones.
- Y1‐5 and its genomic resources are valuable for understanding disease resistance and breeding nematode‐resistant potatoes.

## Plain Language Summary

Potato cyst nematodes (PCNs; *Globodera rostochiensis* and *Globodera pallida*) are serious pests that can cause substantial yield losses in potato crops. Developing potatoes with natural resistance is the most effective and sustainable way to control PCN. In this study, we discovered a wild potato clone, *Solanum brevicaule* (PI 473011) Y1‐5, that shows strong resistance to both PCN species. We subsequently produced a high‐quality reference genome assembly for Y1‐5, which contains over 80,000 predicted protein‐coding genes. Among these are many *NLR* (*nucleotide‐binding and leucine‐rich repeat*) genes, which encode resistance proteins having key roles in helping plants recognize and defend against pests and diseases. This novel Y1‐5 clone, along with its genomic information, provides valuable resources for understanding potato resistance and for breeding new potato varieties with durable resistance, supporting more sustainable potato production.

**Abbreviations**
CNL: coiled‐coil‐type NLR
CPC: Commonwealth Potato Collection
HiFi: high‐fidelity
HMW: high molecular weight
LAI: LTR assembly index
LRR: leucine‐rich repeat
LTR: long terminal repeat
NB: nucleotide‐binding
NLR: nucleotide‐binding and leucine‐rich repeat
PCN: potato cyst nematode
PTI: pattern‐triggered immunity
RS: relative susceptibility
TE: transposable element
TIR: Toll/interleukin‐1 receptor
TNL: TIR‐type NLR
USPG: US Potato Genebank

## 1 INTRODUCTION

Potato (*Solanum tuberosum* L.) is a starchy tuber that originates from the Andean region of South America. It was domesticated over 7000 years ago and has since become a staple food in many parts of the world. Rich in carbohydrates, vitamins, and minerals, potatoes are not only a key source of nutrition but are also incredibly versatile in culinary applications. The adaptability of domesticated potatoes to diverse climates and soils has made them a vital agricultural commodity globally. Today, potatoes are enjoyed in countless dishes, from classic French fries to hearty soups, and play a significant role in food security in many countries. In the United States, potatoes are the leading vegetable crop. The US ranks fifth in global potato production, producing over 440 million hundredweight of potatoes valued at $5.00 billion in 2023 (USDA‐NASS, 2024).

Potato cyst nematodes (PCNs), *Globodera rostochiensis* and *Globodera pallida*, are among the most damaging pests of potatoes and are subject to quarantine regulations in most countries. These nematodes originated in the Andes of South America, where they co‐evolved with their host plants of the genus *Solanum* (Saenz & Scurrah, 1977; Stone, 1985). PCNs were introduced to Europe in the mid‐19th century through tubers imported from South America, and from there they spread to potato‐growing regions worldwide (Brodie & Mai, 1989; Evans et al., 1975; Price et al., 2021). Based on their ability to reproduce on potato genotypes carrying different resistance sources, *G. rostochiensis* and *G. pallida* have been classified into five (Ro1–Ro5) and three pathotypes (Pa1–Pa3), respectively (Kort et al., 1977). In Europe, Ro1 is the most widespread pathotype of *G. rostochiensis* (Evans & Stone, 1977; Gartner et al., 2021), while the predominant *G. pallida* pathotypes are Pa2 and Pa3, which often occur in mixed populations and are generally referred to as Pa2/3 (Dalton et al., 2013).

In the United States, *G. rostochiensis* (pathotype Ro1) was first detected in New York State in 1941. Since then, it has been successfully contained within the state as the result of strict quarantine regulations and the implementation of rotation schemes utilizing Ro1‐resistant potato varieties (Brodie & Mai, 1989; Dandurand et al., 2019). However, the Ro2 pathotype, capable of reproducing on Ro1‐resistant varieties, was discovered in New York in 1995 (Brodie, 1995). This detection has threatened the long‐standing success of *G. rostochiensis* quarantine in New York, as infested fields must be planted with resistant varieties. Yet, to date, only two commercial varieties with Ro2 resistance are available to growers for managing their Ro2‐infested fields (Brodie & Mai, 1989; Dandurand et al., 2019). In 2006, *G. pallida* was detected in Idaho, marking the first detection of a second PCN species in the United States (Hafez et al., 2007). Virulence tests of the Idaho *G. pallida* population revealed a similar virulence pattern to European Pa2/3 (Blok & Phillips, 2012).

The deployment of natural host resistance remains the most effective and environmentally sustainable means for combating crop pathogens, including PCN. In their native origin, PCN coevolved with wild potato species. Early efforts to identify sources of resistance to PCN involved screening numerous wild potato accessions from the Commonwealth Potato Collection (CPC). This led to the discovery of the resistance (R) gene *H1*, found in *S. tuberosum* subsp. *andigena* (CPC 1673), which confers resistance to *G. rostochiensis* pathotypes Ro1 and Ro4 (Ellenby, 1952). The *H1* gene has been widely utilized by breeding programs leading to the release of many Ro1‐resistant potato varieties (Brodie & Mai, 1989; Dandurand et al., 2019; De Jong et al., 2024). The emergence of Ro2 in New York is hypothesized to be a consequence of the continuous cultivation of potato varieties containing *H1* (Brodie, 1995). In Europe, the widespread deployment of *H1* has caused an increase of *G. pallida* populations in potato fields (Dalton et al., 2013).

Over 20 loci conferring resistance to PCN have been identified from various wild potato species, particularly *Solanum spegazzinii*, *Solanum vernei*, and *S. tuberosum* ssp. *andigena* (De Jong et al., 2024; Gartner et al., 2024). Among them, the *Grp1* locus, which confers major resistance to *G. rostochiensis* Ro5 and partial resistance to *G. pallida* Pa2/3 (van der Voort et al., 1998), was recently indicated to be responsible for conferring resistance to *G. rostochiensis* Ro2 found in New York (Park et al., 2019). In the United States, introgression of *Grp1* into breeding germplasm has resulted in the release of two commercial varieties that are resistant to both *G. rostochiensis* pathotypes (Dandurand et al., 2019; De Jong et al., 2024). Breeding for resistance to *G. pallida* has been challenging due to the genetic diversity of *G. pallida* populations and polygenic nature of plant resistance (Dalton et al., 2013; Gartner et al., 2021; Price et al., 2021).

The US Potato Genebank (USPG) maintains a rich collection of wild potato species, including progeny of *S. tuberosum* subsp. *andigena* CPC 1673 (Kiru et al., 2005). However, this valuable resource remained largely unexplored for novel resistance to US PCN populations until recent years. We initially screened several wild species from the USPG, including *S. brevicaule*, and identified a clone (designated Y1‐5) of *Solanum brevicaule* accession PI 473011 that exhibits robust resistance to *G. rostochiensis* pathotypes Ro1 and Ro2, as well as to *G. pallida*. Subsequently, we generated a haplotype‐resolved, reference‐quality genome assembly for Y1‐5, performed functional annotation, and analyzed its repertoire of *NLR* (*nucleotide‐binding and leucine‐rich repeat*) genes encoding nucleotide‐binding (NB) and leucine‐rich repeat (LRR) proteins. Y1‐5 is a diploid species that is sexually compatible with cultivated potatoes (*S. tuberosum*) (Hawkes, 1958; Hawkes & Hjerting, 1969, 1989; Ochoa, 1990). This novel *S. brevicaule* clone and its genomic resources provide a valuable foundation for elucidating the mechanisms of PCN resistance and supporting breeding efforts to develop improved potato varieties with durable nematode resistance.

**Core Ideas**

- A *Solanum brevicaule* clone Y1‐5 was identified to confer robust resistance to potato cyst nematodes.
- A high‐quality, chromosome‐scale reference genome assembly for Y1‐5 was generated.
- The genome comprises 80,021 predicted protein‐coding genes, including a comprehensive set of genes encoding nucleotide‐binding and leucine‐rich repeat (NLR) proteins.
- Y1‐5 encodes more coiled‐coil‐type NLR (CNL) proteins than several previously sequenced wild *Solanum* clones.
- Y1‐5 and its genomic resources are valuable for understanding disease resistance and breeding nematode‐resistant potatoes.

## 2 MATERIALS AND METHODS

### 2.1 Plant materials and screening for resistance against *G. rostochiensis* and *G. pallida*

Botanical seeds of *Solanum brevicaule* accession PI 473011 were obtained from the USPG located in Sturgeon Bay, Wisconsin. *S. brevicaule* PI 473011 was originally collected in Jujuy, Argentina (coordinates: −23.7833, −65.5500) in 1971 and was donated to USPG in 1982 (https://npgsweb.ars‐grin.gov/gringlobal/accessiondetail?id=1367947). PI 473011 underwent a nomenclature name change in 2010 from *Solanum gourlayi* Hawkes subsp. *gourlayi* to *S. brevicaule* Bitter (https://npgsweb.ars‐grin.gov/gringlobal/accessiondetail?id=1367947). True seeds of PI 473011 were surface‐sterilized, germinated, and maintained as individual plantlets (designated Y1 clones) in tissue culture. Tubers derived from each of the Y1 clones were obtained by propagating tissue‐cultured plantlets in soil pots grown under greenhouse conditions at 20°C–23°C with a 12‐h light cycle.

The *G. rostochiensis* Ro1 and Ro2 pathotypes were originally isolated from infested potato fields in New York and have since been maintained in the laboratory through propagations on either an Ro1‐susceptible (lacking the *H1* gene, for Ro1) or an Ro1‐resistant plant (carrying *H1*, for Ro2) (Brodie, 1995; Brodie & Mai, 1989; Wang et al., 2021). *Globodera pallida* was originally isolated from fields in Idaho (Skantar et al., 2007; Whitworth et al., 2018). We initially used a canister bioassay method (Phillips, 1981) to evaluate the resistance of *S. brevicaule* Y1 clones to Ro2. Briefly, a tuber piece containing one to two sprouts from each clone was placed onto moist Cornell soil mix within an 8‐ounce plastic container (consolidated plastics). A water suspension containing approximately 3000 Ro2 eggs was inoculated into three holes around the tuber piece in the container. After inoculation, the container was closed and stored in a dark cardboard box at room temperature (20°C–23°C). Nematode cysts observed through the container wall under a stereoscope were counted 8 weeks after nematode inoculation. Three replicates for each clone were included in the canister bioassay, and clones with no more than five cysts produced were further evaluated for resistance against Ro1, Ro2, and *G. pallida* using pot bioassays across multiple years, as previously described (Park et al., 2018, 2019; Whitworth et al., 2018). Brodie, an Ro2‐resistant variety (De Jong et al., 2024), and Innovator, a European variety with *G. pallida* resistance (Bradshaw, 2009), were included as resistant controls, and varieties Désirée and *S. tuberosum* DM1‐3 516 R44 (referred to as DM1‐3) (The Potato Genome Sequencing Consortium, 2011) were included as susceptible controls. At least three to four replicates for each genotype were included in the pot bioassay, and the final nematode cyst count was obtained for each plant after plant soil was extracted (Brodie, 2003; Park et al., 2019; Whitworth et al., 2018). Désirée or the susceptible DM1‐3 clone was used to calculate relative susceptibility (RS) values as final cyst density of test genotype/final cyst density of Désirée (EPPO, 2006) or DM1‐3 x 100. RS values were then converted to a 0–9 scale (EPPO, 2006), with 0 being the most susceptible and 9 being the most resistant. At least three independent pot bioassay tests were performed, and similar results were obtained.

### 2.2 Sample collection and sequencing

High molecular weight (HMW) DNA isolation, PacBio library preparation, and sequencing were performed at the University of Oregon Genomics and Cell Characterization facility. Approximately 1 g of flash‐frozen leaves from *S. brevicaule* clone Y1‐5 was used for HMW DNA extraction using the Nanobind Plant Nuclei Kit (PacBio). The extracted HMW DNA was sheered into ∼20 kb fragments using a MegaRuptor 2 (Diagenode). PacBio high‐fidelity (HiFi) libraries were prepared with the SMRTbell Express Template Prep Kit 2.0 and the SMRTbell Enzyme CleanUp Kit 2.0 under overnight ligation with non‐barcoded SMRTbell adapters. Libraries were size‐selected to remove <10–12 kb fragments using BluePippin (Sage Science). The long‐read sequencing was performed using two 8 M SMRT cells on the PacBio Sequel II platform, and HiFi subreads generated were post‐processed to obtain final reads with PacBio default parameters (CCS.how). The Hi‐C library was prepared and sequenced using Illumina NextSeq2000 150‐bp paired‐end sequencing technology (Arima Genomics) and the Hi‐C map data were used to scaffold the assembled contigs into a chromosome‐level genome assembly. For transcriptome evidence, 1 cm nodal stem segments from tissue‐culture plants were excised and cultured on propagation medium plates for 3 weeks under a 16‐h light and 8‐h dark cycle at 24°C (Chronis et al., 2014). Roots (excluding root tips) were then collected. Leaves samples were collected from plants grown at greenhouse under a 16‐h light and 8‐h dark cycle for approximately 1 month. Total RNA was extracted from leaf and root tissues using TRIzol (Invitrogen). RNA samples were submitted to Novogene for library preparation and Illumina NovaSeq 6000 150‐bp paired‐end sequencing.

### 2.3 Genome assembly

De novo assembly of PacBio HiFi reads was performed using HiFiasm v0.16 (Cheng et al., 2021). The resulting haplotype‐resolved contigs were used to create a chromosome‐level assembly with Hi‐C scaffolding. Specifically, filtered Hi‐C reads were mapped to both haplotype‐resolved contigs independently using the Arima Genomics mapping pipeline (https://github.com/ArimaGenomics/mapping_pipeline). The resulting data were then used to create chromosome‐level assembly (scaffolds) using the YAHS pipeline (Zhou et al., 2023). Interaction matrix for the scaffolds and heatmaps was generated, and scaffolds were error‐corrected with Juicebox assembly tools v2.17 (Robinson et al., 2018). Assembly completeness was assessed using BUSCO (Benchmarking Universal Single‐Copy Orthologs) v5.4.3 (Manni et al., 2021) with the solanales_odb10 dataset. Genome heterozygosity and size were estimated from HiFi reads using a kmer‐based approach (*k* = 151) with Jellyfish v2.3 (Marçais & Kingsford, 2011) and GenomeScope 2.0 (Vurture et al., 2017). To further evaluate assembly quality, the LTR (long terminal repeat) assembly index (LAI) score was calculated using ltr_harvest v1.6.1 (Ellinghaus et al., 2008), LTR_FINDER_parallel v1.07 (Xu & Wang, 2007), and LTR_retriever (Ou & Jiang, 2018).

### 2.4 Genome annotation

De novo identification of repeat sequences of the assembled genome was performed using RepeatModeler v2.0.4 package (Flynn et al., 2020). The resulting repeat library was combined with the Repbase library (Jurka et al., 2005), which was used to identify repetitive elements in the haplotype‐resolved assembly in soft‐masking mode via RepeatMasker v4.1 (Nishimura, 2000). Gene prediction was conducted in two phases. In the first phase, gene prediction was conducted using BRAKER3 v3.08 (Gabriel et al., 2021, 2024). RNA‐seq reads from Y1‐5 leaf and root tissues were aligned to the genome assembly using HISAT2 v2.2.1 (Kim et al., 2019). The resulting alignments were used to train AUGUSTUS v3.4.0 (Stanke et al., 2006) for the prediction of gene models during BRAKER3 annotation. BRAKER3 annotations then served as a reference for transcriptome assembly with StringTie v2.2.1 (Pertea et al., 2015), which was merged with transcriptomes derived from potato RNA‐seq datasets available in NCBI (PRJNA657265; Zhou et al., 2020). In the second phase, the merged transcriptome, coding sequences from a previous published *S. brevicaule* genome PG5032 (Tang et al., 2022), reviewed homologous plant protein sequences from the UniProt Swiss‐Prot database (https://www.uniprot.org/downloads), published protein sequences from *S. tuberosum* DM6.1 (Pham et al., 2020), and amino acid sequences of NLR proteins from Solanaceae genome sequences (Kourelis et al., 2021) were compiled as evidence for gene prediction using MAKER2 v3.01 (Holt & Yandell, 2011). MAKER2 annotations were iteratively refined in a second run with SNAP (https://github.com/KorfLab/SNAP), which was trained using gene models from the initial MAKER2 run.

The completeness of the predicted gene set was evaluated with BUSCO (Manni et al., 2021) using the solanales_odb10 protein database. Functional annotation of the predicted proteins was performed by BLASTp v2.16.0 (parameters: ‐evalue 1e‐6, ‐max‐target‐seqs 1) searches against UniProt Swiss‐Prot (Boutet et al., 2007) (Release 2026_01), followed by domain and motif annotation using InterProScan v5.76 (P. Jones et al., 2014).

### 2.5 Genome comparison analysis

The genome assembly of *S. brevicaule* Y1‐5 was aligned to the *S. tuberosum* DM6.1 reference genome sequence (Pham et al., 2020) using minimap2 v2.26 (Li, 2018) (‐ax asm5 –eqx) and visualized using D‐GENIES v1.5 (Cabanettes & Klopp, 2018). Structural variations were identified using Syri v1.6.3 (Goel et al., 2019) with the parameters ‐k ‐F S, and the results were visualized using Plotsr v1.1.0 (Goel & Schneeberger, 2022). A second comparative analysis was performed by aligning Y1‐5 haplotype 2 to multiple *Solanum* genome sequences, including *S. tuberosum* DM6.1 (Pham et al., 2020), *Solanum chacoense* (M6; Hamilton et al., 2025), *Solanum verrucosum* (Hosaka et al., 2022), and *Solanum bulbocastanum* (Hosaka et al., 2024), using the same methodology described above.

The predicted gene models from *S. brevicaule* haplotype 2 were compared with those of *S. tuberosum* DM6.1 (Pham et al., 2020), *S. chacoense* (Hamilton et al., 2025), *S. verrucosum* (Hosaka et al., 2022), and *S. bulbocastanum* (Hosaka et al., 2024). Orthologous gene clustering was performed using OrthoVenn3 (Sun et al., 2023), a web‐based platform for comparative genomics. Protein sequences from the analyzed genomes were subjected to all‐versus‐all BLASTp (*E*‐value < 1e‐5), and orthologous gene clusters were defined using OrthoFinder (Emms & Kelly, 2019) with an inflation parameter of 1.5. Phylogenetic evolutionary tree was generated using FastTree2 (Price et al., 2010). Estimation of gene expansion or contraction for common orthogroups of all species was performed with CAFE5 (Mendes et al., 2020).

Genome‐wide synteny analysis was conducted using MCScan (Python version) (Tang et al., 2024). First, protein‐coding genes were aligned using BLASTp (*E*‐value < 1e‐5) to identify homologous gene pairs. The MCScan tool (Wang et al., 2012) was then applied to detect syntenic blocks based on collinear gene arrangements. The results were visualized using the jcvi.graphics.karyotype module (Tang et al., 2024) to generate comparative chromosome plots.

### 2.6 *NLR* gene prediction and analysis

To identify candidate NLR‐encoding genes, we utilized NLR‐Annotator v0.7‐beta (Steuernagel et al., 2020) to scan the genome assembly for proteins containing NB and LRR domains. To improve the accuracy of *NLR* gene prediction, we incorporated protein evidence from an NLR dataset containing 69,849 Solanaceae NLR proteins (Sugihara et al., 2023). This dataset, together with previously assembled transcripts, was used in MAKER2 v3.01 (Holt & Yandell, 2011) to refine *NLR* gene model predictions on the unmasked genome. A total of 1262 gene models encoding NLR proteins were integrated into the existing annotations. Redundant predictions were removed by excluding any genes overlapping with the reannotated NLRs (Tang et al., 2022). For comparative analysis, NLRtracker (Kourelis et al., 2021) was used to identify and classify NLR proteins from the newly sequenced Y1‐5 genome, the *S. tuberosum* DM6.1 genome (Pham et al., 2020), and two previously published genomes of *S. brevicaule* PG5032 (PI 545977) and *S. boliviense* PG5076 (PI 473122) (Tang et al., 2022). Identified NLR proteins were manually validated based on their domain structures. Finally, a genetic map of *NLR* genes was generated using MG2C (Chao et al., 2021).

## 3 RESULTS AND DISCUSSION

### 3.1 Screening for resistance to PCNs

Using the canister bioassay method (Phillips, 1981), our initial screening of several independent clones of *S. brevicaule* accession PI 473011 suggested that among the tested clones, clone Y1‐5 might confer resistance to the *G. rostochiensis* Ro2 pathotype. Subsequent pot bioassays confirmed that Y1‐5 confers robust resistance to *G. rostochiensis* pathotypes Ro1 and Ro2, as well as to *G. pallida*. Based on the European and Mediterranean Plant Protection Organization scale (EPPO, 2006), Y1‐5 received a resistance score of 9 against both *G. rostochiensis* pathotypes and a score of 8 against *G. pallida* (Table 1). Under the same testing conditions, the resistant variety Brodie scored 8 or 7 against *G. rostochiensis* Ro2, while the European variety Innovator scored 8 or 6 against *G. pallida* (Table 1). The Y1‐5 clone is diploid and closely related to *S. tuberosum*. Given its broad‐spectrum resistance to PCN, Y1‐5 represents a valuable germplasm resource for the cloning of novel resistance genes and for breeding potatoes with durable PCN resistance.

**TABLE 1:** Evaluation of *Solanum brevicaule* (PI 473011) Y1‐5 for resistance to *Globodera rostochiensis* Ro1 and Ro2 pathotypes and *G. pallida*.

Test year	Clone	Average cyst number/pot	Relative susceptibility (RS) (%)^a^	Score^b^
***G. rostochiensis* Ro1 bioassay tests**				
2020	Y1‐5	0	0	9
	DM1‐3	775		
2021	Y1‐5	0	0	9
	Désirée	355		
2023	Y1‐5	0	0	9
	Désirée	228		
***G. rostochiensis* Ro2 bioassay tests**				
2020	Y1‐5	0	0	9
	DM1‐3	668		
2021	Y1‐5	0	0	9
	DM1‐3	597		
2021	Y1‐5	0	0	9
	Brodie	12	3.23	7
	DM1‐3	372		
2023	Y1‐5	0	0	9
	Désirée	309		
2024	Y1‐5	0	0	9
	Brodie	11	1.99	8
	Désirée	554		
***G. pallida* bioassay tests**				
2021	Y1‐5	4	1.23	8
	DM1‐3	324		
2022	Y1‐5	5	1.95	8
	Désirée	256		
2023	Y1‐5	26	2.65	8
	Innovator	55	5.6	6
	Désirée	982		
2024	Innovator	7	1.16	8
	Désirée	603		
2025	Innovator	14	1.9	8
	Désirée	736		

^a^Relative susceptibility = final cyst density of test genotype/final cyst density of Désirée or DM1‐3 x 100.

^b^Scores 9 (being the highest level of resistance), 8, 7,6 correspond to RS of <1%, 1.1%–3%, 3.1%–5%, and 5.1%–10%, respectively (EPPO, 2006).

### 3.2 Genome assembly and gene prediction

**FIGURE 1:**
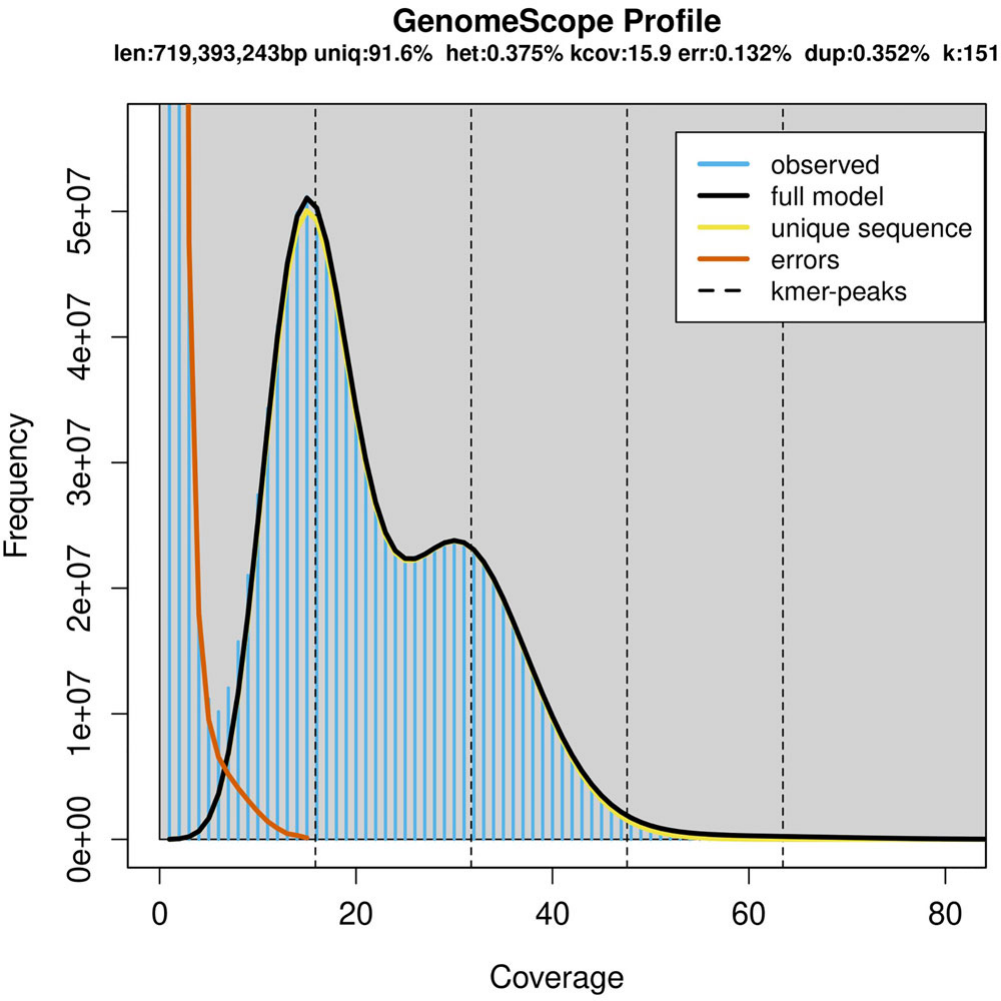
Genome size estimation of PacBio high‐fidelity (HiFi) reads using Jellyfish and Genomescope v2.0. The plot showed two major peaks for the diploid genome of *Solanum brevicaule* (PI 473011) Y1‐5.

PacBio HiFi sequencing produced 30.6 Gb of final reads, corresponding to ∼36.9x coverage. Estimated genome size based on the *k*‐mer (*k* = 51) is 756 Mb with a heterozygosity of 0.667% (Figure 1 and Figure S1). Potato genomes vary widely in size and ploidy, ranging from diploids to hexaploids, with genome sizes of ∼700–800 Mb. Our assembly size is consistent with those of other reported wild diploids, including *Solanum verucosum* (780.2 Mb), *Solanum okadae* (728.98 Mb), and *Solanum commersonii* (∼830 Mb) (Achakkagari et al., 2024; Aversano et al., 2015; Hosaka et al., 2024). The observed variation in estimated genome sizes between Y1‐5 and other reported *Solanum* species is likely due to natural variation rather than technical limitations of the assembly.

**FIGURE 2:**
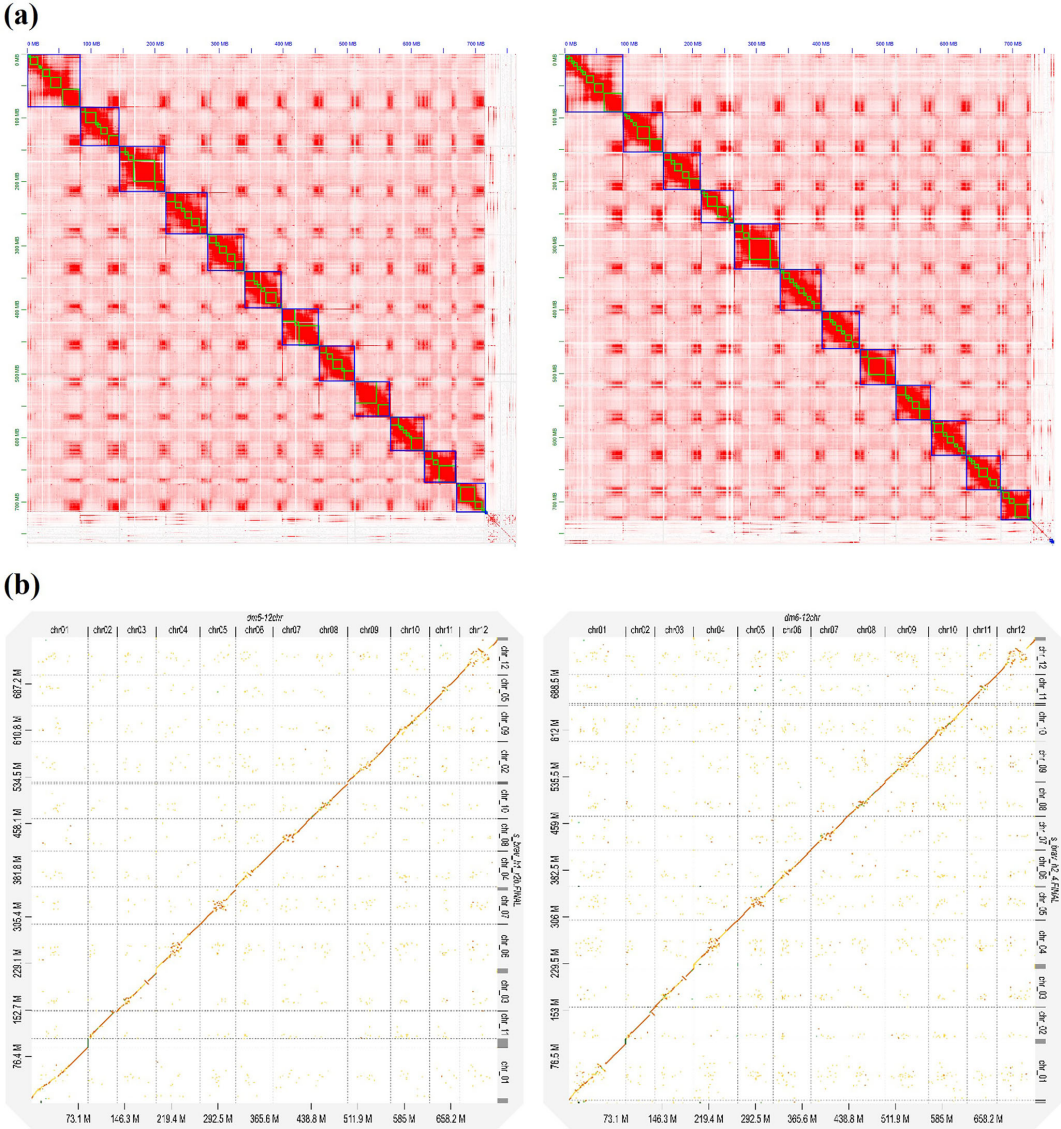
Hi‐C map and chromosome alignment of *Solanum brevicaule* Y1‐5 haplotype 1 and haplotype 2 chromosome‐level assembly. (a) Interaction matrix of Y1‐5 haplotype 1 and haplotype 2 generated with Hi‐C reads using the YAHS pipeline and Juicebox tools. (b) Comparison of haplotypes 1 and 2 of Y1‐5 with the *S. tuberosum* DM6.1 assembly.

At the contig level, the HiFi‐based assembly consisted of 1068 contigs for haplotype 1 and 507 contigs for haplotype 2, with maximum contig lengths of 34.9 and 33.5 Mb, respectively (Table 2). Arima Hi‐C and Illumina HiSeq sequencing yielded 518 million 150‐bp paired‐end reads. After mapping these reads to the initial contig‐level assembly and scaffolding with the YAHS pipeline and Juicer tools, the final scaffold numbers were reduced to 975 for haplotype 1 and 392 for haplotype 2 (Table 2; Figure 2a). The final chromosome‐level assembly produced genome sizes of 763.5 and 764.9 Mb for haplotypes 1 and 2, respectively, with N50 scaffold lengths of 57.9 and 59.5 Mb (Table 2). The 12 chromosomes contained 716.3 and 729.4 Mb in the final assembly for haplotypes 1 and 2, respectively. Unplaced contigs accounted for 47.2 and 35.6 Mb, corresponding primarily to repetitive sequences, organellar genomes, and transposable elements (TEs), consistent with previous reports (Hosaka et al., 2024; Sharma et al., 2022; Sun et al., 2022). Chromosome orientation and order were corrected through alignment with the *S. tuberosum* DM6.1 reference genome (Figure 2b) (Pham et al., 2020).

**TABLE 2:** Assembly statistics of *Solanum brevicaule* (PI 473011) Y1‐5 after scaffolding with Hi‐C, YAHS pipeline, and Juicer tools.

	**Haplotype 1**	**Haplotype 2**
Main genome scaffold total	975	392
Main genome contig total	1068	507
Main genome scaffold sequence total	763.504 Mbp	764.949 Mbp
Main genome scaffold L50	6	6
Main genome contig L50	18	22
Main genome scaffold N50	57.925 Mbp	59.502 Mbp
Main genome contig N50	15.275 Mbp	11.764 Mbp
Main genome scaffold L90	12	12
Main genome contig L90	55	68
Main genome scaffold N90	45.29 Mbp	46.552 Mbp
Main genome contig N90	2.832 Mbp	2.787 Mbp
Max scaffold length	83.307 Mbp	91.371 Mbp
Max contig length	34.949 Mbp	33.525 Mbp

BUSCO analysis indicated genome completeness of 96.6% for haplotype 1 (93.7% single copy, 2.9% duplicated, 0.2% fragmented, and 3.2% missing) and 98.1% for haplotype 2 (93.1% single copy, 5% duplicated, 0.2% fragmented, and 1.7% missing) (Table 3). LAI is a metric to evaluate the quality of genome assemblies with respect to the intactness of LTR retrotransposons, with higher LAI scores reflecting better assembly quality (LAI < 10: draft‐level assembly; LAI = 10–20: improved assembly; and LAI > 20: reference‐quality assembly). LAI analysis indicated completeness of 8.19 and 13.47 for haplotypes 1 and 2, respectively. Both BUSCO and LAI values were comparable to those reported for high‐quality potato assemblies such as *S. tuberosum* DM6.1 (LAI = 13.56), *S. verrucosum* (LAI = 11.97), and *S. bulbocastanum* (LAI = 9.63) (Hosaka et al., 2024; Pham et al., 2020), indicating a reference‐quality, haplotype‐resolved genome assembly of the *S. brevicaule* Y1‐5 clone.

**TABLE 3:** Benchmarking universal single‐copy orthologs (BUSCO) completeness analysis of *Solanum brevicaule* (PI 473011) Y1‐5 genome assembly and structural annotation.

	**Haplotype 1—Assembly**	**Haplotype 2—Assembly**	**Whole genome protein annotation**
Complete	96.6% (5744)	98.1% (5837)	96.2% (5724)
Single copy	93.7% (5574)	93.1% (5542)	2.0% (121)
Duplicated	2.9% (170)	5.0% (295)	94.2% (5603)
Fragmented	0.2% (10)	0.2% (10)	1.4% (84)
Missing	3.2% (196)	1.7% (103)	2.4% (142)
Total Busco's	5950	5950	5950

Abbreviation: BUSCO, benchmarking universal single‐copy orthologs.

The two‐phased gene prediction with BRAKER3 and MAKER2, supported by RNA and protein evidence, identified 80,021 protein‐coding genes (39,644 and 40,377 protein‐coding genes in haplotypes 1 and 2, respectively). The average transcript length was 6141 bp, with a mean exon length of 218 bp and an average of five exons per transcript. BUSCO analysis of the predicted genes against the solanales_odb10 database indicated genome annotation completeness of 96.2% (Table 3). Among these, duplicated genes represented 94.2% across both haplotypes; fragmented and missing genes accounted for 1.4% and 2.4%, respectively, indicating a high‐quality genome assembly and accurate gene prediction. Of the 80,021 protein‐coding genes, 55.1% have functional annotation based on the UniProt Swiss‐Prot protein database, and 54.6% are annotated with PFAM domains.

### 3.3 Synteny and phylogenetic analysis

**FIGURE 3:**
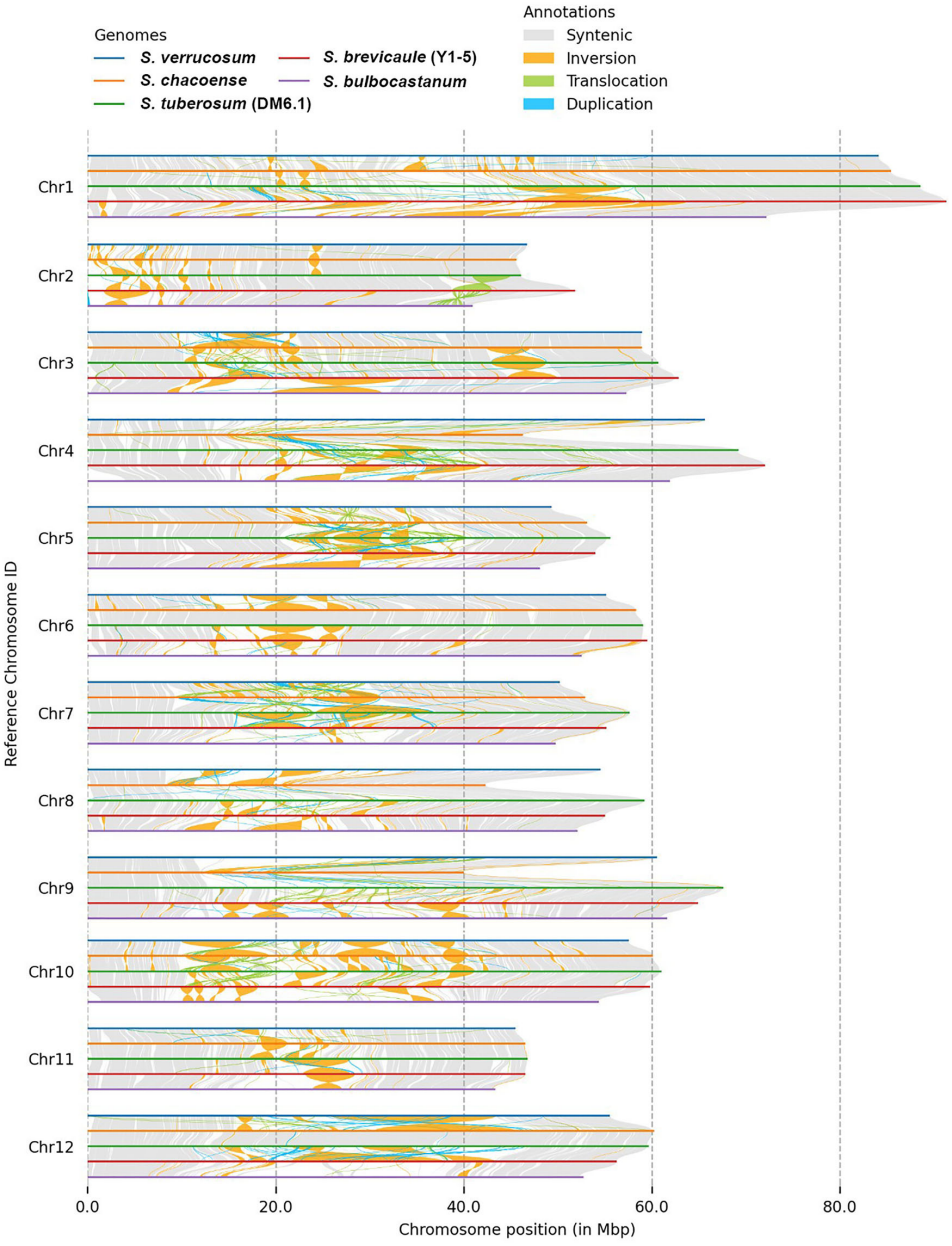
Genome synteny plot of *Solanum brevicaule* (PI 473011) Y1‐5 with other *Solanum* species,, including *S. tuberosum* (DM6.1), *S. verrucosum*, *S. chacoense*, and *S. bulbocastanum*.

Dotplot comparison between genome assemblies of *S. brevicaule* Y1‐5 and *S. tuberosum* DM6.1 using D‐GENIES showed that approximately 50% of the Y1‐5 sequences shared about 50% identity with the DM6.1 genome (Figure 2b). Analysis of chromosome synteny between the haplotype‐resolved Y1‐5 assembly and the DM6.1 genome revealed extensive synteny regions across all 12 homologous chromosome groups. The chromosomes showed end‐to‐end alignment with no substantial regions of misalignment (Figure S2). These results demonstrated that the Y1‐5 genome assembly is highly complete and meets the criteria for chromosome‐level resolution. The pattern of conservation in sub‐telomeric regions compared to other parts of the chromosomes is shown in Figure 3. Similar patterns were also reported in other wild *Solanum* species (Hosaka et al., 2022, 2024; D. Tang et al., 2022).

**FIGURE 4:**
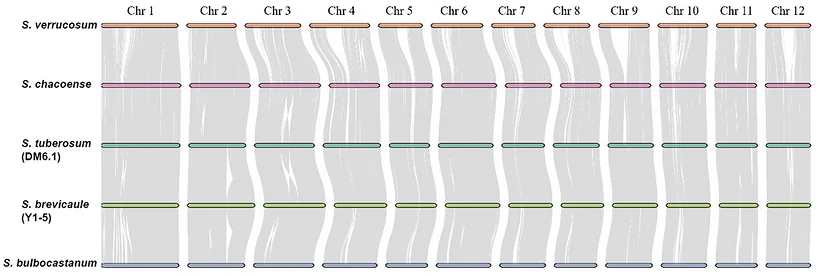
Gene synteny of *Solanum brevicaule* (PI 473011) Y1‐5 with other *Solanum* species including *S. tuberosum* (DM6.1), *S. verrucosum*, *S. chacoense*, and *S. bulbocastanum*.

**FIGURE 5:**
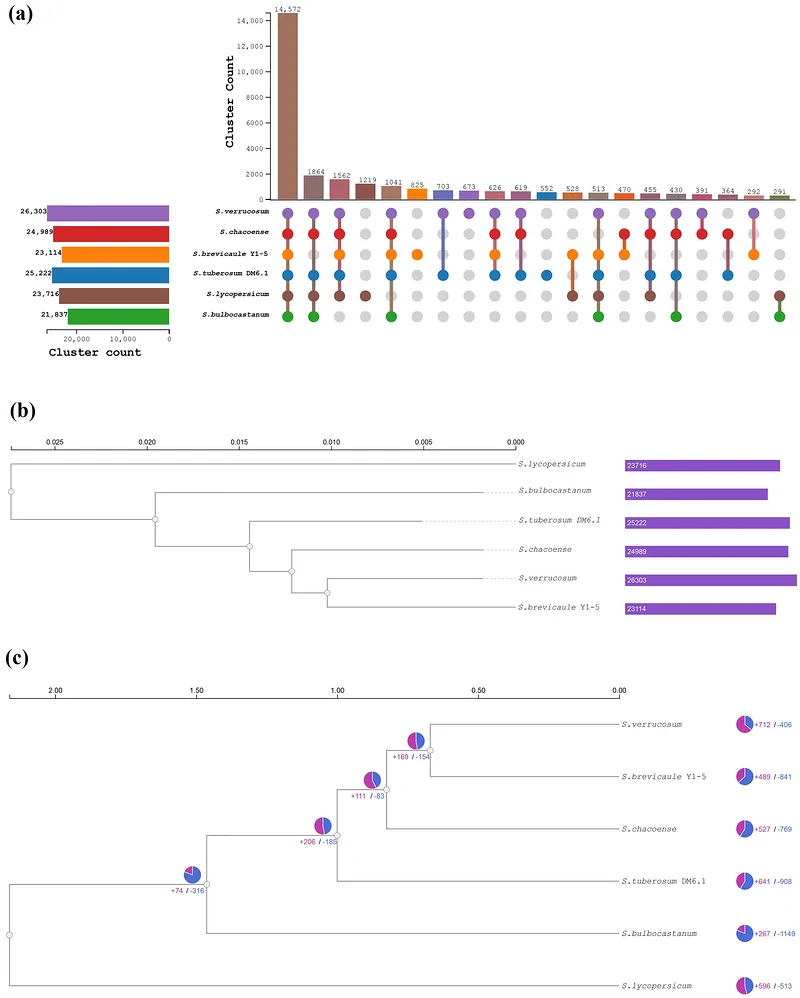
Phylogenetic analysis of *Solanum brevicaule* (PI 473011) Y1‐5 and four other potato species using OrthoVenn3. (a) UpSet plot comparison of shared orthogroups between Y1‐5 and other *Solanum* species, including *S. tuberosum* (DM6.1), *S. verrucosum*, *S. chacoense*, and *S. bulbocastanum*. (b) Orthofinder‐generated phylogenetic tree constructed using 14,572 common orthogroups among Y1‐5 and the five other *Solanum* species. (c) Expansion and contraction of common orthogroups observed in Y1‐5 and other *Solanum* species.

Within the Y1‐5 genome, Syri analysis identified high‐level synteny between the two haplotypes, with 393 syntenic regions covering 623 Mb (86.64%) of the 12 anchored chromosomes. Inversions between haplotypes 1 and 2 were identified on chromosomes 1, 3, and 9 consistent with patterns reported by Hosaka et al. (2024) (Figure 3). Comparative analysis of Y1‐5 haplotype 2 with other *Solanaceae* genome sequences, including *S. tuberosum* DM6.1, *S. verrucosum*, *S. chacoense*, and *S. bulbocastanum*, revealed broad chromosome‐level synteny across these species except for *S. bulbocastanum*, a Mexican B‐genome species, which exhibited lower synteny with Y1‐5 (Figures 3 and 4). Inversions were predominantly observed on different regions of chromosomes among these species (Figure 3). Inversions represent a major mode of chromosomal differentiation in potatoes and tomatoes (Gaiero et al., 2021). These structural variations play critical roles in maintaining co‐adapted alleles, enabling wild plant populations to preserve advantageous genetic combinations that enhance their adaptation to specific environmental conditions (Wellenreuther & Bernatchez, 2018). In *Solanum*, inversions have likely facilitated adaptation to diverse ecological niches, as reflected by distinct loci targeted during the domestication of upland and lowland potato populations (Hardigan et al., 2017). Inversions may also contribute to the maintenance of genetic diversity and adaptive potential (Hardigan et al., 2017). Understanding the mechanisms underlying these chromosomal inversions may offer valuable insights into their evolutionary significance and inform breeding strategies to improve resilience in cultivated potatoes. Synteny plot analysis revealed extensive collinear gene blocks across the five Solanaceous species (*S. brevicaule* PI 473011, *S. bulbocastanum*, *S. chacoense*, *S. tuberosum*, and *S. verrucosum*). The observed synteny between the *S. tuberosum* reference genome DM6.1 and the other wild potato species is consistent with previous reports indicating that potato genomes have undergone relatively limited chromosomal rearrangements (Figure 4; Hardigan et al., 2017). However, notable rearrangements of syntenic blocks were detected on chromosomes 1, 2, and 3 of *S. brevicaule*, *S. bulbocastanum*, and *S. verrucosum*. Similarly, an upset plot analysis using OrthoVenn3 identified 14,572 orthogroups shared among the species studied (Figure 5a), further supporting evidence of their common evolutionary origin. Y1‐5 contained 23,114 gene clusters, while higher numbers of gene clusters were observed in *S. verrucosum* (26,303) and *S. tuberosum* DM6.1 (25,222) (Figure 5a). The variation in gene cluster numbers among these potato species suggests potential species‐specific expansions and contractions, which may have contributed to their adaptation to diverse environmental niches.

Phylogenetic analysis using tomato (*Solanum lycopersicum* SL5) as the outgroup supported the established evolutionary relationships among *Solanum* species. As expected, *S. lycopersicum* was placed as an outgroup in the phylogenetic tree generated by OrthoVenn3 (Figure 5b). *S. bulbocastanum*, a Mexican B‐genome species, diverged earlier than the other A‐genome species including Y1‐5, *S. tuberosum*, *S. chacoense*, and *S. verrucosum* (Figure 5b), consistent with previous report (Hosaka et al., 2024; D. Tang et al., 2022). Notably, *S. verrucosum* was found to be most closely related to *S. brevicaule* Y1‐5 (Figure 5b). Gene expansion and contraction analysis revealed substantial gene gains for the branch (+169 genes) of *S. brevicaule* and *S. verrucosum*, and the branch (+111 genes) of *S. brevicaule*, *S. verrucosum*, and *S. chacoense*, with less gene loss for those branches (Figure 5c). This potentially reflects evolutionary adaptations such as pathogen resistance or tolerance to environmental stresses (Hosaka et al., 2022). Conversely, the large‐scale gene losses detected in *S. brevicaule* (−841) and *S. bulbocastanum* (−1149) may represent processes of genome streamlining or pseudogenization events.

### 3.4 Repeat modeling

RepeatModeler and RepeatMasker were used to identify and mask repetitive elements in the Y1‐5 genome. Repeat elements accounted for 511.15 Mb (66.95%) and 502.26 Mb (65.66%) of haplotypes 1 and 2, respectively (Table 4). In comparison, repeat elements generally constitute approximately 50%–60% of the genome in other Solanaceous species (Mehra et al., 2015). The proportion observed in this study is slightly higher than that reported in other wild potato genomes (Achakkagari et al., 2024).

**TABLE 4:** Repeat analysis of haplotypes 1 and 2 of the *Solanum brevicaule* (PI 473011) Y1‐5 genome using RepeatModeler and RepeatMasker tools.

	**Haplotype 1**			**Haplotype 2**		
	**Number of elements**	**Length (bp)**	**Percentage of sequence**	**Number of elements**	**Length (bp)**	**Percentage of sequence**
Retroelements	229,655	258,708,546	33.88	234,846	261,282,718	34.16
SINEs	2525	1,865,973	0.24	2520	935,641	0.12
LINEs	46,363	21,930,449	2.87	47,291	19,586,738	2.56
R1/LOA/Jockey	2138	3,521,339	0.46	2022	852,826	0.11
RTE/Bov‐B	20,610	4,966,727	0.65	21,086	5,068,225	0.66
L1/CIN4	23,615	13,442,383	1.76	24,183	13,665,687	1.79
LTR elements	180,767	234,912,124	30.77	185,035	240,760,339	31.47
Ty1/Copia	35,283	27,432,209	3.59	36,241	28,197,951	3.69
Gypsy/DIRS1	137,102	190,823,617	24.99	140,596	196,638,682	25.71
Retroviral	95	49,041	0.01	107	52,619	0.01
DNA transposons	37,397	17,076,510	2.24	38,517	17,338,050	2.27
hobo‐Activator	10,180	4,730,127	0.62	10,413	4,752,589	0.62
Tc1‐IS630‐Pogo	2915	780,319	0.10	3022	803,559	0.11
Tourist/Harbinger	6307	2,322,391	0.30	6497	2,374,285	0.31
Rolling‐circles	4304	2,981,516	0.39	4094	2,690,869	0.35
Unclassified	610,668	206,552,097	27.05	625,037	203,303,982	26.58
Total interspersed repeats	–	482,337,153	63.17	–	481,924,750	63.00
Small RNA	10,447	18,751,608	2.46	9116	10,423,065	1.36
Satellites	57	58,696	0.01	51	43,259	0.01
Simple repeats	108,677	5,776,428	0.76	111,334	5,874,703	0.77
Low complexity	20,896	1,247,987	0.16	21,747	1,304,699	0.17
		Total	66.95		Total	65.66

**FIGURE 6:**
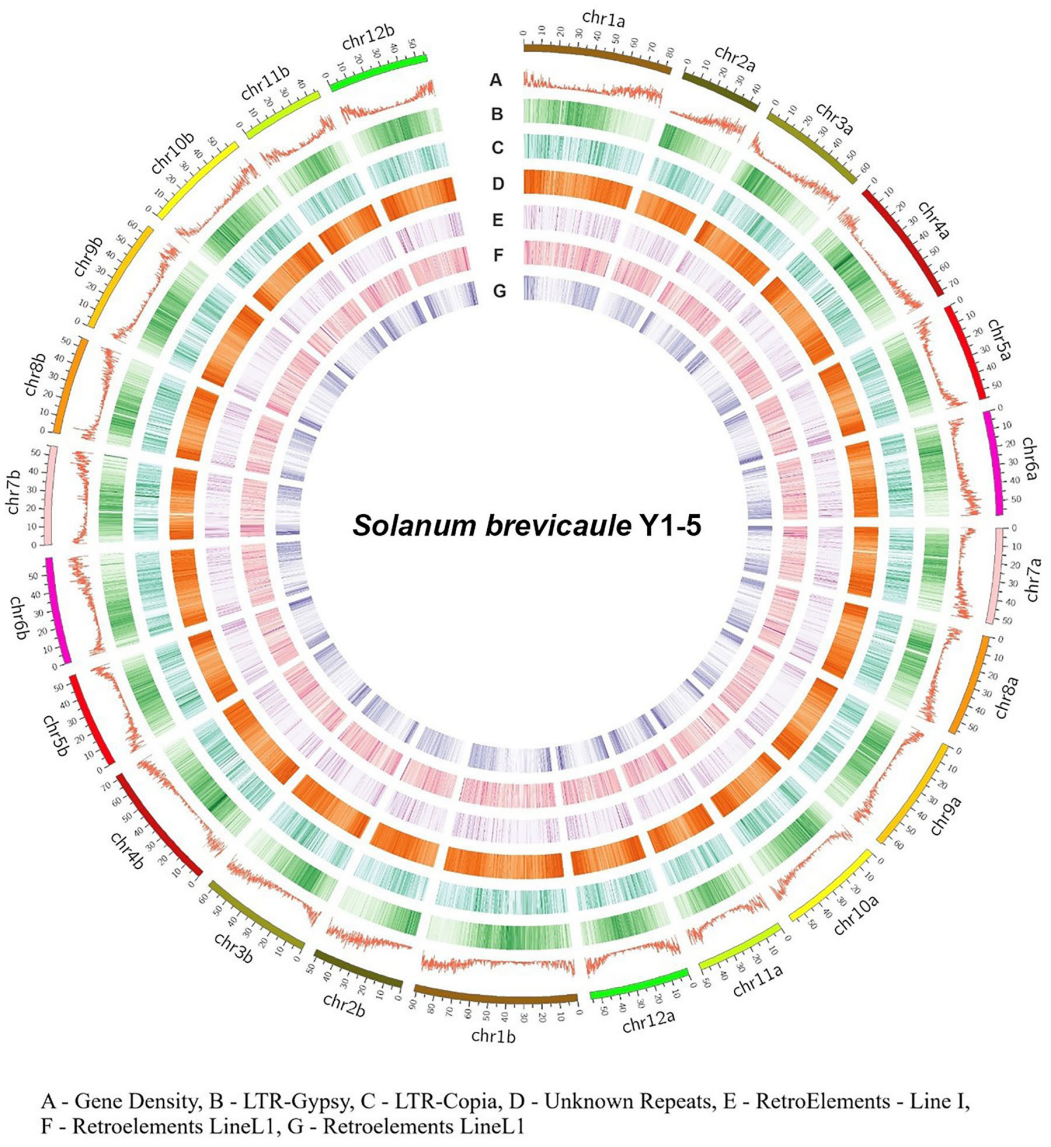
Chromosomal distribution of genes and repetitive elements across all 24 chromosomes of *Solanum brevicaule* (PI 473011) Y1‐5.

Repetitive elements play an important role in the genomes of Solanaceous species, particularly for *S. tuberosum* (potato). These elements, including TEs and other various repeat types, have contributed to genome expansion and structural evolution. TEs constitute a significant portion of *Solanum* genomes, including those of potato and tomato (Gantuz et al., 2021; Grover & Sharma, 2017). RNA TE, or retroelements, were found to represent a significant fraction, accounting for 33.88% and 34.16% of haplotype 1 and haplotype 2, respectively (Table 4; Figure 6). These values are consistent with earlier reports showing retrotransposons as key contributors to genome size and diversity in *Solanum* (Gantuz et al., 2021; Merkulov et al., 2024). Among retroelements, LINE (long interspersed nuclear elements) elements comprised 2.87% and 2.56% of haplotype 1 and haplotype 2, while LTR elements accounted for 30.77% in haplotype 1 and 31.47% in haplotype 2 (Table 4; Figure 6). Among LTR elements, Gypsy elements constituted 24.99% (haplotype 1) and 25.71% (haplotype 2), making them more abundant than Copia elements, which accounted for 3.59% (haplotype 1) and 3.69% (haplotype 2) (Table 4; Figure 6). This Gypsy > Copia abundance pattern has also been documented in other Solanaceous genomes (de Assis et al., 2020; Esposito et al., 2019). DNA TEs were present at similar levels in both haplotypes, accounting for 2.24% (37,397 elements) (haplotype 1) and 2.27% (38,517 elements) (haplotype 2), with the hobo‐Activator and Tourist/Harbinger families being the most prevalent (Table 4; Figure 6). Unclassified elements also represented a significant portion of the genome (27.05% in haplotype 1 and 26.58% in haplotype 2), contributing to total interspersed repeat contents of 63.17% in haplotype 1 and 63.00% in haplotype 2 (Table 4; Figure 6). Small RNA elements, simple repeats, and low‐complexity regions were similarly distributed across both haplotypes (Table 4). In summary, the Y1‐5 genome is highly enriched in repeat elements, particularly retrotransposons, consistent with other Solanaceous species. These findings underscore the important roles of repetitive sequences in shaping genome architecture, driving structural variation, and influencing the evolutionary dynamics of this plant family.

### 3.5 *NLR* gene prediction

In the plant immune system, the first line of defense involves the recognition of conserved pathogen‐associated molecular patterns, which activates basal immune responses known as pattern‐triggered immunity (PTI) (J. D. G. Jones & Dangl, 2006). In addition to PTI, plants employ intracellular receptors often known as disease resistance (R) proteins, that directly or indirectly detect specific pathogen effectors to turn on effector‐triggered immunity (J. D. G. Jones & Dangl, 2006). Most R genes encode NLRs receptors, which are broadly classified into coiled‐coil (CC)‐type NLRs (CNLs) and Toll/interleukin‐1 receptor (TIR)‐type NLRs (TNLs) (J. D. G. Jones & Dangl, 2006; Ngou et al., 2022).

Computational annotation tools such as BRAKER (Hoff et al., 2019) and MAKER (Cantarel et al., 2008) often underpredict *R* genes due to several factors, including their tissue‐specific expression (Lüdke et al., 2025), the masking of *R* gene regions during repeat filtering (Bayer et al., 2018), and their frequent clustering or duplication within plant genomes (Andolfo et al., 2014; Jupe et al., 2012, 2013). To overcome these challenges, specialized pipelines such as NLR‐annotator scan all six reading frames of genomic sequences for conserved NLR motifs, enabling more comprehensive detection of candidate *NLR* loci. Using this approach, we identified 886 and 861 putative *NLR*‐gene loci in haplotypes 1 and 2, respectively.

**FIGURE 7:**
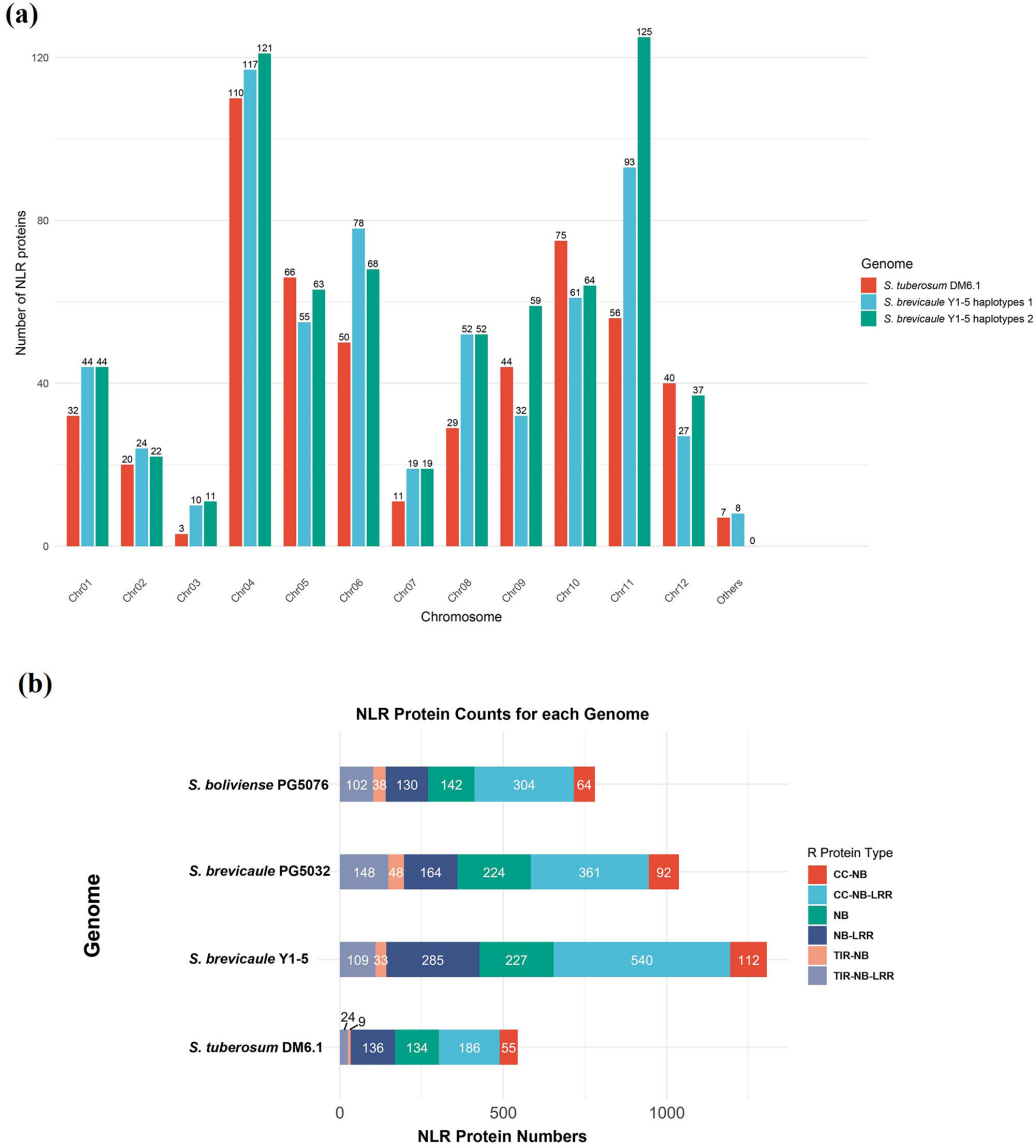
Comparison of *nucleotide‐binding and leucine‐rich repeat* (*NLR*) genes encoding nucleotide‐binding (NB) domain in *Solanum brevicaule* (PI 473011) Y1‐5 and other potato genomes. (a) Genetic map of *NLR* genes identified by NLRtracker on all 24 chromosomes of Y1‐5 haplotypes 1 and 2. (b) Distribution of different types of *NLR* genes identified by NLRtracker in Y1‐5 and previous published genomes of *S. tuberosum* DM6.1, *S. brevicaule* PG5032 (PI 545977), and *S. boliviense* PG5076 (PI 473122).

In the annotated Y1‐5 genome sequence, we identified 1306 NB domain‐encoding genes, all of which were manually inspected for domain structure. Those genes were enriched on chromosomes 4, 5, 6, 8, 10, and 11, each harboring over 50 *NLR*s (Figure 7a). The difference in *NLR* gene copy numbers observed between the two haplotypes may reflect either assembly‐related sequence losses or genuine structural variations between homologous chromosomes. Among the 1306 genes, 540 are classified as CNLs (CC, NB, and LRR), 109 as TNLs (TIR, NB, and LRR), 112 as CNs (CC and NB), 285 as NLs (NB and LRR), 227 as NBs, and 33 as TNs (TIR and NB) (Figure 7b). We further constructed a genetic map of *NLR*s across the 24 chromosomes of haplotypes 1 and 2, highlighting *CNL* and *TNL* genes (Figure 8). The map revealed that most *NLR*s occur in clusters within localized chromosomal regions. Notably, the clustering of *CNL* and *TNL* genes was especially pronounced on chromosomes 4, 5, 10, and 11, suggesting that these regions may represent genomic hotspots with critical roles in disease resistance.

FIGURE 8Genetic map of *nucleotide‐binding and leucine‐rich repeat* (*NLR*) gene on all 24 chromosomes of *S. brevicaule* (PI 473011) Y1‐5 haplotype 1 (a) and haplotype 2 (b). Genes encoding CNL (CC‐NB‐LRR) proteins are highlighted in red, while genes encoding TIR‐type NLR (TNL) (TIR‐NB‐LRR) proteins are highlighted in blue.

**Figure:**
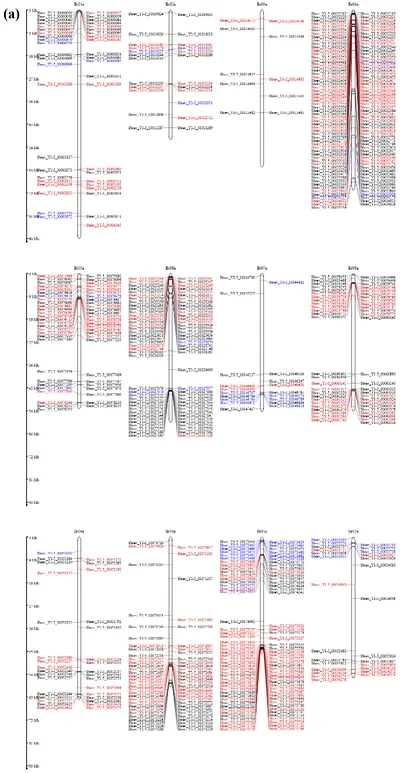

**Figure:**
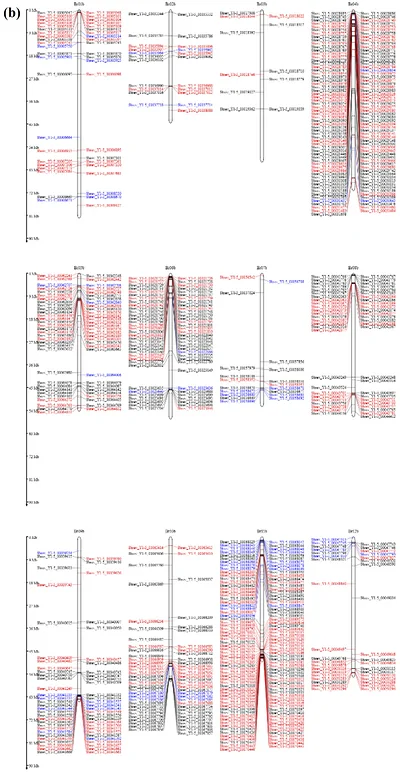

Previous research has shown variation in *NLR* gene copy numbers among different potato species (Tang et al., 2022). To ensure comparability, we applied the same *NLR*‐gene annotation pipeline to the Y1‐5 genome assembly, two previously published genome sequences of *S. brevicaule* PG5032 (PI 545977) and *S. boliviense* PG5076 (PI 473122), and the *S. tuberosum* DM6.1 reference genome sequence (Tang et al., 2022). *Solanum boliviense* was indicated to be a promising wild *Solanum* species for screening novel PCN resistance (Castelli et al., 2003). Interestingly, Y1‐5 was found to encode more NLRs than the other three genomes, including *S. brevicaule* PG5032 (Figure 8b). Moreover, Y1‐5 appeared to harbor a greater number of *CNL*s than *S. brevicaule* PG5032 and *S. boliviense* PG5076, as well as several other previously sequenced wild *Solanum* species (Figure 8b; Supplementary Table 6 of Tang et al., 2022). To date, three PCN resistance genes have been cloned, all belonging to the NLR family (Ernst et al., 2002; Paal et al., 2004; van der Vossen et al., 2000). The potato *Gpa2* and the tomato *Hero* genes encode CNL proteins, while the potato *Gro1‐4* gene encodes a TNL protein. Other genes conferring resistance to root‐knot nematodes, including the tomato *Mi‐1* gene and the two pepper (*Capsicum annuum* L.) genes *CaRKNR* and *CaMi*, have been cloned, all encoding CNL proteins (Chen et al., 2007; Mao et al., 2015; Vos et al., 1998). The enrichment of CNL‐encoding genes in Y1‐5 suggests potential novel sources of resistance against PCN and other soil‐borne pests and pathogens.

## 4 CONCLUSION

In this study, we identified the *S. brevicaule* (PI 473011) Y1‐5 clone as highly resistant to both *G. rostochiensis* and *G. pallida*. We generated a haplotype‐resolved, referenced‐quality genome for Y1‐5, which demonstrated high completeness and contiguity, as evidenced by strong BUSCO and LAI scores, making it comparable to existing potato reference genomes. This assembly has helped enrich current potato genomic resources.

Our analyses revealed that Y1‐5 harbors repeat content typical of Solanaceous species and retains high levels of chromosomal and gene synteny with other *Solanum* species. Gene annotation identified 80,021 putative protein‐coding genes, including 1306 NB domain‐encoding genes. These resources provide a foundation for elucidating the molecular mechanisms underlying resistance to PCN and represent valuable genomic tools for breeding durable resistance in potatoes. Importantly, Y1‐5 clone is sexually compatible with cultivated potatoes (*S. tuberosum*) (Hawkes, 1958; Hawkes & Hjerting, 1969, 1989; Ochoa, 1990), enabling the direct introgression of its resistance traits into breeding lines.

## AUTHOR CONTRIBUTIONS

**Senthilkumar Shanmugavel**: Data curation; formal analysis; methodology; writing—original draft; writing—review and editing. **Shengwei Hu**: Data curation; formal analysis; methodology; writing—original draft; writing—review and editing. **Huijun Yang**: Data curation; formal analysis; methodology; writing—review and editing. **Shiyan Chen**: Formal analysis; writing—review and editing. **John Bamberg**: Resources; writing—review and editing. **Dongyan Zhao**: Methodology; writing—review and editing. **Bhoja R. Basnet**: Writing—review and editing. **Craig T. Beil**: Supervision; writing—review and editing. **Moira J Sheehan**: Funding acquisition; writing—review and editing. **Vidyasagar Sathuvalli**: Funding acquisition; supervision; validation; writing—review and editing. **Xiaohong Wang**: Conceptualization; funding acquisition; project administration; resources; supervision; writing—original draft; writing—review and editing.

## CONFLICT OF INTEREST STATEMENT

The authors declare no conflicts of interest.

## Supporting information




**FIGURE S1** Genome size estimation of PacBio HiFi reads using Jellyfish and Genomescope v2.0 with k‐mer sizes of k = 21 (a), k = 51 (b), k = 101 (c), and k = 151 (d).

**FIGURE S2** Comparison of haplotype 1 and 2 chromosomes of *Solanum brevicaule* (PI 473011) Y1‐5 with *S. tuberosum* DM6.1.

## Data Availability

The raw reads and assembly can be accessed from the NCBI database with project id—PRJNA1218720. Public Transcriptome data from NCBI used for the functional annotation of the *Solanum brevicaule* genome can be found in *Solanum brevicaule* (leaf, shoot)—SRR23225939, SRR23225938, and *S. tuberosum* (young and mature leaves, stems, stolons, and tubers)—SRR14298414, SRR14298447, SRR14298454, SRR14298455, SRR14298444, SRR14298437, SRR14298435, SRR14298419, and SRR14298420.
