# Supplementary figures and images for: A chromosome‐level genome assembly of Solanum brevicaule (PI 473011) Y1‐5, a wild potato relative with robust resistance to potato cyst nematodes
Source: Plant Genome. 2026 Jun 12;19(2):e70265. doi: 10.1002/tpg2.70265 (PMC13261804; doi:10.1002/tpg2.70265)

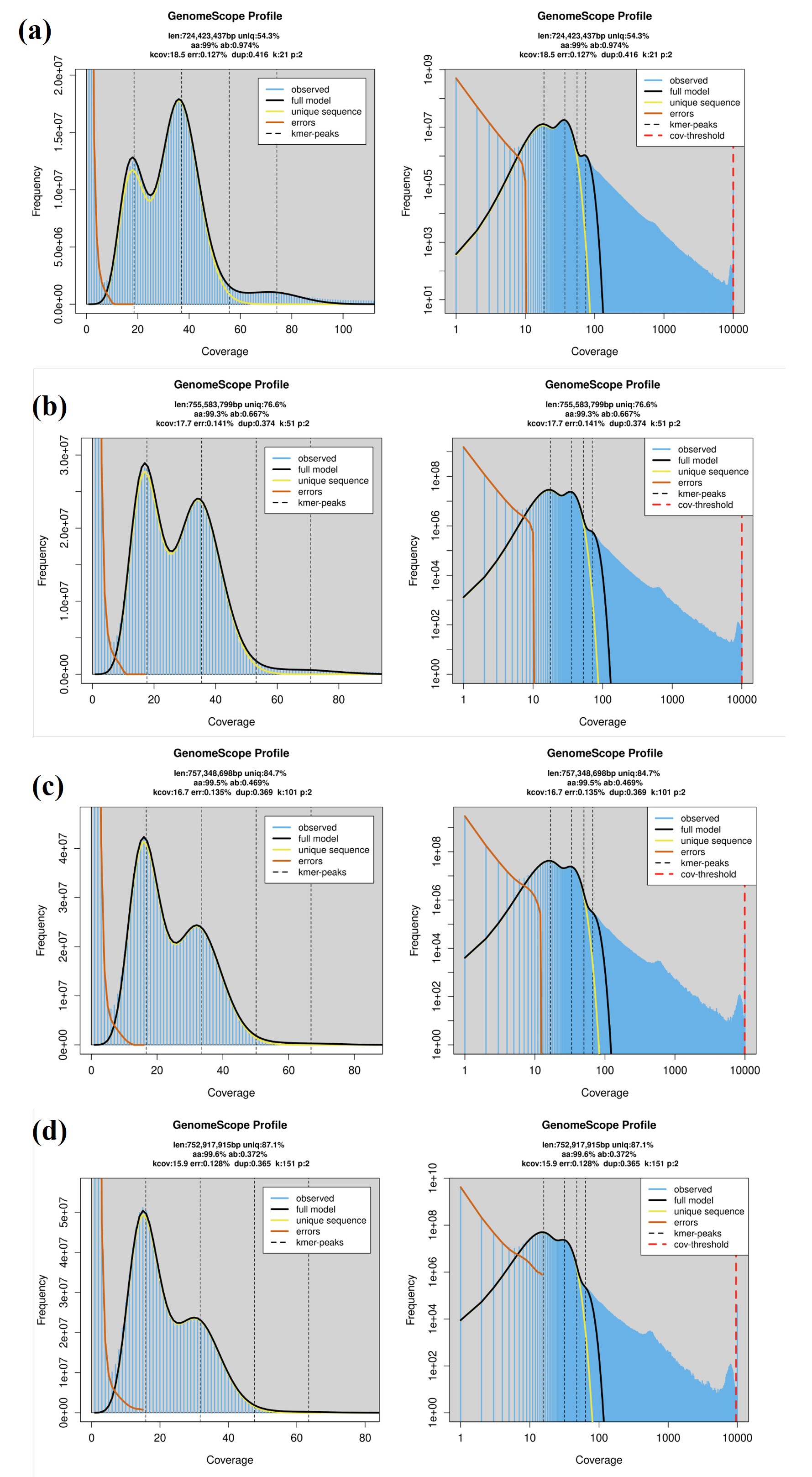

Supplement: Supplementary file 1 — FIGURE S1 Genome size estimation of PacBio HiFi reads using Jellyfish and Genomescope v2.0 with k‐mer sizes of k = 21 (a), k = 51 (b), k = 101 (c), and k = 151 (d). [file TPG2-19-e70265-s001.jpg]

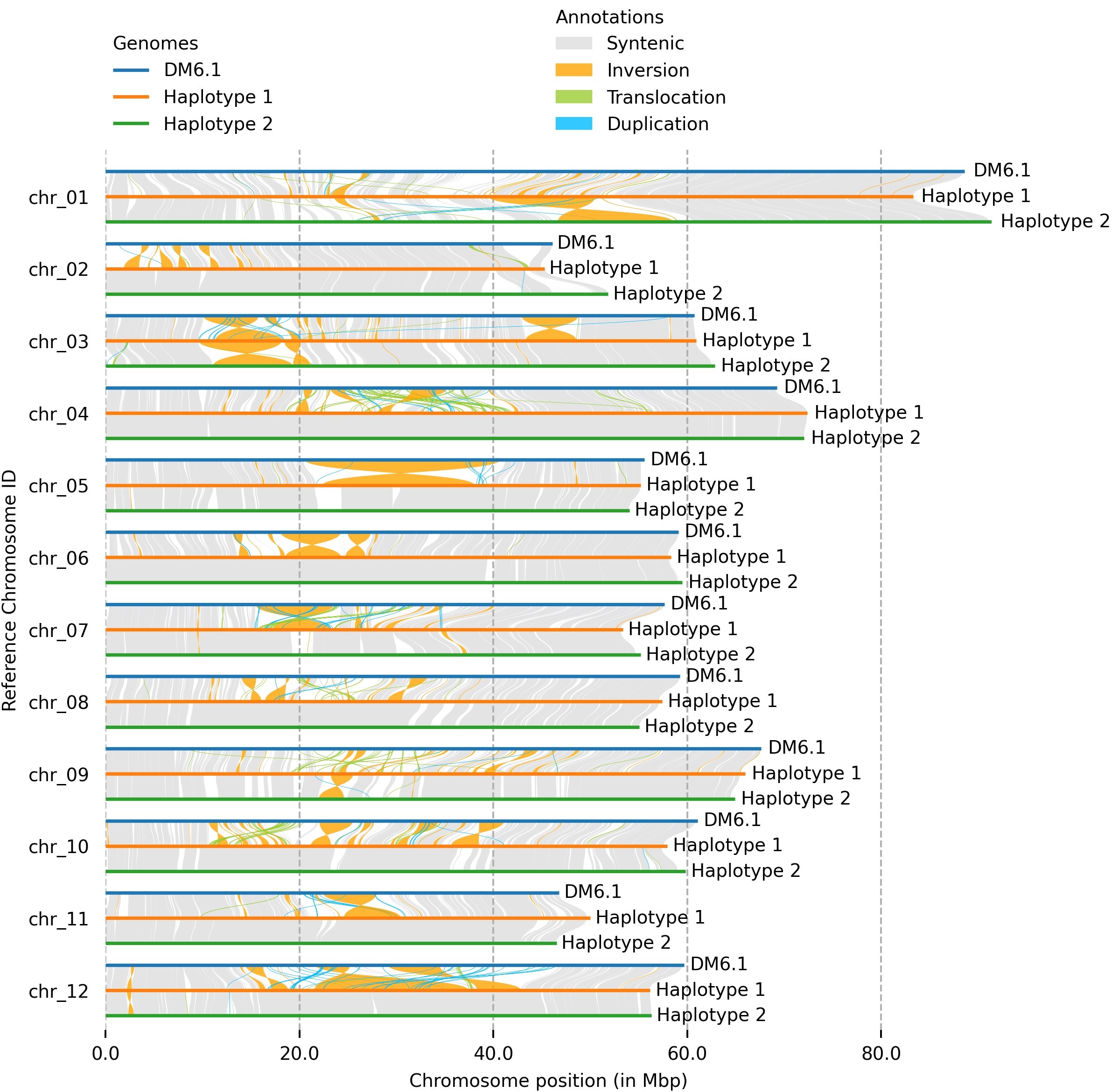

Supplement: Supplementary file 2 — FIGURE S2 Comparison of haplotype 1 and 2 chromosomes of Solanum brevicaule (PI 473011) Y1‐5 with S. tuberosum DM6.1. [file TPG2-19-e70265-s002.jpg]
